# Dynamic personalized risk prediction in chronic heart failure patients: a longitudinal, clinical investigation of 92 biomarkers (Bio-SHiFT study)

**DOI:** 10.1038/s41598-022-06698-3

**Published:** 2022-02-18

**Authors:** Dominika Klimczak-Tomaniak, Marie de Bakker, Elke Bouwens, K. Martijn Akkerhuis, Sara Baart, Dimitris Rizopoulos, Henk Mouthaan, Jan van Ramshorst, Tjeerd Germans, Alina Constantinescu, Olivier Manintveld, Victor Umans, Eric Boersma, Isabella Kardys

**Affiliations:** 1 Department of Cardiology, Erasmus MC, University Medical Center Rotterdam, Room NA-316, P.O. Box 2040, 3000 CA Rotterdam, The Netherlands; 2 Department of Cardiology, Hypertension and Internal Medicine, Medical University of Warsaw, Warsaw, Poland; 3 Department of Biostatistics, Erasmus MC, University Medical Center Rotterdam, Rotterdam, The Netherlands; 4 Olink Proteomics AB, Uppsala, Sweden; 5 Department of Cardiology, Northwest Clinics, Alkmaar, The Netherlands

**Keywords:** Cardiology, Prognostic markers

## Abstract

The aim of our observational study was to derive a small set out of 92 repeatedly measured biomarkers with optimal predictive capacity for adverse clinical events in heart failure, which could be used for dynamic, individual risk assessment in clinical practice. In 250 chronic HFrEF (CHF) patients, we collected trimonthly blood samples during a median of 2.2 years. We selected 537 samples for repeated measurement of 92 biomarkers with the Cardiovascular Panel III (Olink Proteomics AB). We applied Least Absolute Shrinkage and Selection Operator (LASSO) penalization to select the optimal set of predictors of the primary endpoint (PE). The association between repeatedly measured levels of selected biomarkers and the PE was evaluated by multivariable joint models (mvJM) with stratified fivefold cross validation of the area under the curve (cvAUC). The PE occurred in 66(27%) patients. The optimal set of biomarkers selected by LASSO included 9 proteins: NT-proBNP, ST2, vWF, FABP4, IGFBP-1, PAI-1, PON-3, transferrin receptor protein-1, and chitotriosidase-1, that yielded a cvAUC of 0.88, outperforming the discriminative ability of models consisting of standard biomarkers (NT-proBNP, hs-TnT, eGFR clinically adjusted) − 0.82 and performing equally well as an extended literature-based set of acknowledged biomarkers (NT-proBNP, hs-TnT, hs-CRP, GDF-15, ST2, PAI-1, Galectin 3) − 0.88. Nine out of 92 serially measured circulating proteins provided a multivariable model for adverse clinical events in CHF patients with high discriminative ability. These proteins reflect wall stress, remodelling, endothelial dysfunction, iron deficiency, haemostasis/fibrinolysis and innate immunity activation. A panel containing these proteins could contribute to dynamic, personalized risk assessment.

Clinical Trial Registration: 10/05/2013 https://clinicaltrials.gov/ct2/show/NCT01851538?term=nCT01851538&draw=2&rank=1.

## Introduction

A broad array of biological pathways contributes to the development and progression of heart failure (HF), including neuro-hormonal and remodeling mechanisms^1^. In addition to being a syndrome encompassing activation of cardiovascular disease-related mechanisms, heart failure may also be viewed as a systemic disorder with alterations to metabolism, immune response, haemostasis/fibrinolysis, and iron homeostasis^2–4^. In the past two decades the ability has emerged to measure circulating proteins, related to abovementioned underlying pathophysiological mechanisms, to monitor processes in HF patients that reflect and predict adverse changes before they become clinically apparent. This ability gives them the potential to detect progression and increased risk in an earlier stage compared to traditional physiological markers such as symptoms, blood pressure and weight, which appear late in the time course of decompensation. Proteins whose use for risk stratification is currently recommended by the guidelines include natriuretic peptides, biomarkers of myocardial injury (cardiac troponins) and biomarkers of myocardial fibrosis (ST2 and galectin-3)^5,6^. However, room for improvement of risk stratification still remains.

Previous studies on circulating biomarkers carry two important limitations. First, they have usually examined one or a few biomarkers at the time. There are only few examples of a more comprehensive metabolomic or proteomic assessment performed with the use of mass spectrometry^7,8^ or aptamer-based and proximity extension proteomic assays in the context of heart failure^9,10^, which could help us improve our understanding of the diverse biologic pathways activated during heart failure. Second, previous studies on circulating proteins have traditionally performed measurements at study baseline and relate them to adverse events occurring over many years thereafter; or have left several years in between (usually two) repeated measurements^1,8–10^. To the best of our knowledge, no study so far has assessed an elaborate set of circulating proteins in the longitudinal setting, nor used repeatedly applied multiplex panels to construct clinical prediction models in heart failure patients.

Within The Serial Biomarker Measurements and New Echocardiographic Techniques in Chronic Heart Failure Patients Result in Tailored Prediction of Prognosis (Bio-SHIFT) study, we repeatedly evaluated 92 protein biomarkers in a clinically stable population of chronic HF (CHF) patients over 2.2 years of follow-up. We have first explored subsets of individual proteins in the context of the pathophysiological pathways they contribute to, including inflammation, fibrinolysis, cardiac remodeling and endocrine regulation, and found that a large proportion of circulating proteins involved in these pathways are individually associated with adverse clinical events^11,12^. With the current analysis we aim to derive a set of protein biomarkers, selected from the repeatedly measured comprehensive panel of 92, that is most informative for prognostication.

### Hypothesis and purpose

We hypothesize that applying a penalization method to the comprehensive set of repeatedly measured biomarkers will lead to a dynamic prediction model that takes into account the various biological mechanisms involved but is at the same time sufficiently lean for application in clinical practice.

## Methods

### Study design

The Bio-SHiFT Study is a prospective cohort study of stable patients with CHF, conducted in Erasmus MC, Rotterdam, and Noordwest Ziekenhuisgroep, Alkmaar, Netherlands, registered in ClinicalTrials.gov (NCT01851538). The study design has been described in detail previously^13^. From October 2011 onwards, consecutive patients were screened. We included ambulatory adult patients if CHF had been diagnosed according to European Society Guidelines at least 3 months before, and the clinical course was stable (i.e. they had not been hospitalized for HF in the past 3 months). The design of the Bio-SHiFT study has been described in detail previously. Exact inclusion and exclusion criteria are presented in Supplemental Fig. S1. The study was approved by the appropriate medical ethics committees (MEC-2011-029 by Medisch Ethische Toetsings Commissie Erasmus MC, and M011-024 by Medisch Ethische Toetsingscommissie Noord-Holland), and conducted in accordance with the Declaration of Helsinki. All the included patients signed informed consent. Analyses presented in this article comprised 250 heart failure with reduced ejection fraction (HFrEF) patients selected out of 263 patients with CHF enrolled during the first inclusion period (October 2011 until June 2013). Neither patients nor the public were involved in the research.

### Baseline assessment and follow-up procedures

At the inclusion and during follow-up visits, all patients were evaluated by research physicians, who were blinded for biomarker results. Routine outpatient visits were performed by the treating physicians. Study follow-up visits were predefined and scheduled every 3 months (± 1 month), in parallel to routine visits at the outpatient clinic. A clinical event committee, blinded to the biomarker-candidate results, reviewed hospital records and discharge letters and adjudicated the study endpoints. No patient was lost to follow-up.The primary endpoint (PE) was a composite of cardiac death, heart transplantation, LVAD implantation, and hospitalization for the management of acute or worsened HF, whichever occurred first. Detailed definitions are described in Text S1 of the Supporting information. Details of baseline assessment and follow-up procedures has been described previously^12^.

### Blood sampling

Blood samples were collected at baseline and at each trimonthly study follow-up visit, and were processed and stored at − 80 °C within two hours after collection until batchwise biomarker measurement was performed after completion of follow-up. In the first inclusion round of the Bio-SHiFT study which we used for the current investigation, we collected a total of 1984 samples before occurrence of the PE or censoring in 263 patients [median (IQR) 9 (5–10) blood samples per patient]. We focused on the 250 patients with HFrEF. For reasons of efficiency, for the current retrospective investigation, we made a selection of 537 samples in these patients: we selected all baseline samples, the last sample available in patients in whom the PE did not occur during follow-up, and the two samples available closest in time prior to the PE (which, by design, were 3 months apart). Our previous analyses using all available samples in this cohort have demonstrated that the concentration of several plasma and urine biomarkers changes over the months preceding the incident adverse event^14^. By selecting the last 2 samples prior to the incident endpoint, we aimed to capture the changes in biomarkers in patients with incident events. Conversely, in event-free patients, our previous investigations showed stable biomarker levels, in which case 1 additional sample suffices. All laboratory personnel were blinded for clinical data and patient outcomes.

### Laboratory measurements

The Cardiovascular panel III of the Olink Multiplex platform (Olink Proteomics AB, Uppsala, Sweden), based on proximity extension assay technology, was used for analysis of 92 high-abundance proteins (listed in Text S2), chosen based on their potential to represent various aspects of cardiovascular pathophysiology. Four internal controls and two external controls were included in each assay. In a validation study, the mean intra-assay and inter-assay coefficients of variation were 8% and 12%, respectively^15^. The biomarker values are delivered in Normalized Protein Expression (NPX) Units, which are relative units that result from the polymerase chain reaction. They are expressed on a log2 scale so that a one unit higher NPX value represents a doubling of the measured protein concentrations. The methods of measurement of the standard biomarkers [N-terminal prohormone brain natriuretic peptide (NT-proBNP), high sensitivity troponin T (hs-TnT) and high sensitivity C-reactive (hs-CRP)] are described in Text S3.

### Statistical analysis

In order to allow for direct comparisons between different biomarkers we used the z score (i.e., the standardized form) of the biomarker levels delivered in log2 of NPX units. Power calculation and missing data handling are described in Text S4. First, we explored biomarker levels at baseline and at the last moment before occurrence of the event or censoring. For this purpose, heatmaps were used to visualize the hierarchical clustering of the biomarkers in patients with the primary endpoint and those who remained endpoint-free.

Then, we took into account temporal evolution of the biomarkers and performed a penalized time-dependent Cox regression with all 92 of the repeatedly measured proteins, in order to select the subset of proteins that carries the best predictive value for the PE and, at the same time, to reduce the risk of overfitting. We used the penalization method LASSO (Least Absolute Shrinkage and Selection Operator), which shrinks the regression coefficients towards zero, resulting in more accurate predictions when the model is applied to new patients^16^. For the selection of the optimum penalty term (λ), we performed 20 replications of a tenfold cross-validation of the model and selected the median value of the obtained penalty terms (equal to 11.2), which provided a model with 9 biomarkers.

Subsequently, we evaluated the associations between the repeated measurements of the 9 biomarkers selected in the previous step and the PE with a multivariable joint model (mvJM). MvJM combines linear mixed effects (LME) models for repeated measurements of multiple biomarkers with a time-to-event relative risk model for the time-to-event data^17^. We used (1) unadjusted mvJMs and (2) mvJMs with both the relative risk and LME (the fixed part) models adjusted for (a) age and sex; (b) systolic blood pressure, NYHA class, duration of CHF (years), diabetes mellitus, baseline NT-proBNP, baseline hs-TnT. The selection of these six clinical covariates was based on the observation that those variables differed significantly in patients in whom the endpoint occurred compared to endpoint-free patients (Table S1). Only those variables were selected that differed significantly after a Benjamini–Hochberg procedure with false discovery rate (FDR) equal to 0.05. Additionally, we evaluated models consisting of biomarkers used in everyday practice (repeatedly measured NT-proBNP, hs-TnT eGFR) as well as a literature-based selection of biomarkers. Sampling time was included in both the fixed and random part of the LME models. Additionally, we constructed corresponding models that included both level and slope of the biomarkers and the PE.

The time-dependent area under the curve (AUC) methodology as adapted for JM was used in order to determine the longitudinal markers’ prospective accuracy^18^. We performed a stratified fivefold cross-validation of these AUCs. Additionally, to evaluate whether adding biomarkers improves the clinical risk model we calculated net reclassification index (NRI)^19^.

Finally, we performed a sensitivity analysis using only the first and last sample both in patients in whom the PE occurred and in patients that remained free of the PE, in order to verify whether using 2 samples in PE-patients vs. 1 sample in non-PE patients may have introduced bias in the selection of proteins and statistical modeling.

Spearman correlation coefficients were calculated to evaluate correlations between baseline values of proteins selected for the final models as well as the correlations between subsequent measurements of biomarkers.

All analyses were performed with R Statistical Software v. 3.4.1. using packages ‘simputation’, ‘penalized’, ‘nlme’, ‘survival’, ‘JMbayes’, ‘riskRegression’, and ‘nricens’.

## Results

Mean age was 67 (58–76) years, 184 (74%) were men, 62 (25%) in NYHA class III-IV and mean left ventricular ejection fraction (LVEF) was 31 ± 9% (Table 1).

**Table 1:** Baseline characteristics of the study population (N = 250).

Variable	
**Demographics**	
Age (years)	67 (58–76)
Male gender	184 (74%)
**Clinical characteristics**	
BMI (kg/m^2^)	27 (24–30)
Systolic blood pressure (mmHg)	120 (108–132)
Diastolic blood pressure (mmHg)	72 (62–80)
Pulse (beats/min)	67 (60–74)
eGFR* (ml/min/1.73 m^2^)	58 (42–76)
**Features of HF**	
LVEF, %	31 ± 9
NYHA class III or IV	62 (25%)
Duration of CHF (years)	4.8 (1.8–9.4)
**Etiology of HF**	
Ischemic heart disease	116 (46%)
Secondary to valvular disease	10 (4%)
Cardiomyopathy	63 (25%)
Other or unknown	61 (24%)
**Medical history**	
Myocardial infarction	95 (38%)
PCI	81 (32%)
CABG	42 (17%)
AF	97 (39%)
Hypertension	113 (45%)
Diabetes mellitus	77 (31%)
Known hypercholesterolemia	94 (38%)
**Biomarker concentrations**	
NT-proBNP (pmol/l)	133 (45–274)
hs-TnT (ng/l)	17.7 (9.3–32.7)
CRP (mg/l)	2.2 (0.9–4.9)
**Biomarkers analyzed within JM**^**†**^	
ST2	3.35 (2.86–3.74)
vWF	6.33 (5.52–7.06)
FABP4	5.95 (5.25–6.74)
IGFBP1	3.38 (1.35)
PAI-1	5.43 (4.76–6.06)
TfR	4.51 (0.69)
CHIT1	6.07 (5.17–6.82)
PON3	5.14 (0.70)
GDF-15	5.57 (0.93)
Gal3	5.26 ± 9.46
**Medication use**	
Beta-blocker	225 (90%)
ACE-I or ARB	235 (94%)
Aldosterone antagonist	72 (29%)
Diuretic	227 (91%)
Loop diuretics	226 (90%)
Thiazides	6 (2%)

Categorical variables are expressed as count (percentage). Values of continuous variables are expressed as mean ± standard deviation or as median (interquartile range) in case of skewed distribution.

*ACE-I* Angiotensin-converting enzyme inhibitor, *ARB* Angiotensin II receptor blocker, *CABG* Coronary artery bypass grafting, *CHIT1* Chitotriosidase-1, *COPD* Chronic obstructive pulmonary disease, *DBP* Diastolic blood pressure, *Gal3* Galectin 3, *GDF-15* Growth/differentiation factor 15, *FABP4* Fatty acid-binding protein 4, *hs-TnT* High-sensitivity troponin T, *IGFBP-1* Insulin-like growth factor-binding protein 1, *KDOQI* Kidney disease outcomes quality initiative, *LVEF* Left ventricular ejection fraction, *MI* Myocardial infarction, *NT-proBNP* N-terminal prohormone of brain natriuretic peptide, *PAI-1* Plasminogen activator inhibitor 1, *PCI* Percutaneous coronary intervention, *PON3* Paraoxonase 3, *SBP* Systolic blood pressure, *ST2* Suppressor of tumorigenicity 2, *TfR* Transferrin receptor protein 1, *vWF* von Willebrand factor.

*Estimated with CKD-EPI equation.

^†^Biomarker concentrations in this table are given in arbitrary NPX (relative) units on a log2 scale, measured at baseline.

During a median (interquartile range) follow‐up of 2.2 (1.4–2.5) years, a total of 29 patients died (among them 24 died from cardiovascular disease). In total 66 (26%) patients reached the composite PE; the first event that occurred was rehospitalization for acute or worsened HF in 53 patients, heart transplantation in 3, underwent left ventricular assist device placement in 2, and death from cardiovascular causes in 8 patients. Median (IQR) time between baseline and the last sample was 1.34 (0.77; 1.73) years for patients in whom PE occurred and 1.97 (1.59; 1.84) years for patients without PE.

Hierarchical clustering of all 92 biomarkers revealed that among patients in whom the endpoint occurred, already at baseline, a much larger proportion of patients had elevated biomarker levels compared to patients that remained endpoint-free, as depicted in the heatmap (Fig. S2). This was observed for most of the proteins, which is in line with the systemic nature of heart failure. This contrast was even greater for the last biomarker measurements preceding the endpoint or censoring (Fig. S3). Additionally, univariable JM analyses of all 92 individual biomarkers demonstrated that levels of most of the proteins were significantly associated with the occurrence of the PE (Table S2).

Penalized time-dependent Cox regression using the repeated measurements of the 92 biomarkers resulted in a selection of 9 biomarkers: NT-proBNP, ST2, von Willebrand factor (vWF), fatty acid-binding protein 4 (FABP4), Insulin-like growth factor-binding protein 1 (IGFBP1), plasminogen activator inhibitor 1 (PAI-1), transferrin receptor protein 1 (TfR), chitotriosidase-1 (CHIT1), and paraoxonase 3 (PON3), implying that for optimal prognostic performance, a model containing these 9 repeatedly measured biomarkers suffices. The sensitivity analysis consisting of the penalized time-dependent Cox regression using only 2 samples in both the PE and non-PE patients resulted in a penalty term (λ) equal to 11.58 and exactly the same selection of 9 biomarkers, including NT-proBNP, ST2, vWF, FABP4, IGFBP1, PAI-1, TfR, CHIT1 and PON3. The HRs and CIs from this analysis are presented in Table S3, and the AUCs are presented in Table S4.

Figures S4 and S5 show the results of hierarchical clustering of the 9 biomarkers selected in the penalization procedure for the baseline and the last measurement, respectively. Similar to the 92-biomarker heatmaps, a contrast between patients with the PE vs. endpoint-free patients can be observed, again more pronounced for the last measurement.

**Figure 1:**
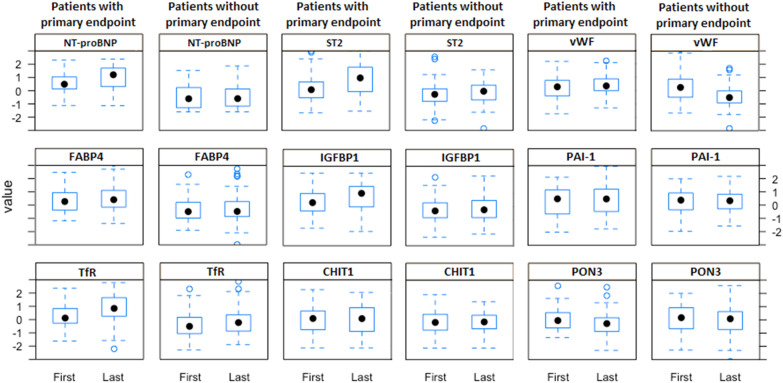
Boxplot figures presenting baseline and last available measurements of biomarkers retained in the model by the LASSO analysis in 250 patients with the endpoint and those who remained endpoint free. *CHIT1* Chitotriosidase-1, *FABP4* Fatty acid-binding protein 4, *IGFBP-1* Insulin-like growth factor-binding protein 1, *NT-proBNP* N-terminal prohormone brain natriuretic peptide, *PAI-1* Plasminogen activator inhibitor 1, *PON3* Paraoxonase 3, *ST2 protein* Suppressor of tumorigenicity 2, *TfR* Transferrin receptor protein 1, *vWF* von Willebrand factor.

More detailed insights into the biomarker values can be obtained from Fig. 1, which presents the boxplots for baseline and last available measurements of these 9 biomarkers in patients with the PE and in those who remained endpoint-free. In patients with an incident PE, the levels of NT-proBNP, ST2, vWF, FABP4, IGFBP1, PAI-1, TfR, and CHIT1 were numerically higher than levels in endpoint-free patients, and this pertained to both the baseline measurement and the last available measurement. Conversely, PON3 level was numerically lower in patients with the PE at both time-points.

When the 9 LASSO-derived biomarkers were subsequently entered into the mvJM, 2 of the 9 biomarkers were statistically significantly associated with the occurrence of primary composite endpoint: NT-proBNP (hazard ratio (HR), [95% confidence interval, (95% CI)]: 1.79 [1.35; 2.45], *p* < 0.001) and vWF 3.21 [1.23; 9.44], *p* = 0.02) as presented in Table 2 (Model 3). Associations remained statistically significant after further adjustment for clinical characteristics (Model 3B, Table S5).

**Table 2:** Multivariable models predicting clinical outcome in the study population (N = 250 patients).

Model 1 Baseline biomarkers and clinical covariates			Model 2 Serially measured established biomarkers adjusted for clinical covariates^‡^			Model 3 Serially measured biomarkers selected from the proteomic panel based on penalized regression (LASSO)			Model 4 Serially measured biomarkers selected from the proteomic panel based on previous literature		
Variable	HR (95% CI)	*p* value	Variable	HR (95% CI)	*p* value	Variable	HR (95% CI)	*p* value	Variable	HR (95% CI)	*p* value
SBP*	0.94 (0.82; 1.07)	0.34	NT-proBNP^†^	2.69 (2.01; 3.63)	< 0.001	NT-proBNP^†^	1.79 (1.35; 2.45)	< 0.001	NT-proBNP^†^	1.70 (1.29; 2.31)	< 0.001
NYHA III or IV	1.48 (0.88; 2.48)	0.14	hs-TnT^†^	1.31 (0.86; 2.09)	0.17	ST2	0.78 (0.45; 1.42)	0.39	hs-TnT^†^	1.26 (0.88; 1.78)	0.19
Duration of CHF	1.06 (1.02; 1.10)	0.004	eGFR^†^	1.63 (0.97; 3.04)	0.06	vWF	3.21 (1.23; 9.44)	0.02	hsCRP^†^	1.26 (0.92; 1.76)	0.17
DM	1.42 (0.85; 2.38)	0.18				FABP4	1.06 (0.68; 1.64)	0.78	GDF-15	1.02 (0.54; 1.97)	0.93
NT-proBNP^†^	1.66 (1.34; 2.05)	< 0.001				IGFBP1	1.23 (0.68; 2.52)	0.54	ST2	1.19 (0.70; 2.04)	0.52
hs-TnT^†^	1.24 (0.95; 1.63)	0.11				PAI-1	1.11 (0.65; 1.85)	0.69	PAI-1	0.98 (0.59; 1.65)	0.97
						TfR	1.37 (0.85; 2.25)	0.20	Gal3	1.37 (0.87; 2.23)	0.16
						CHIT1	0.84 (0.59; 1.20)	0.30			
						PON3	0.92 (0.65; 1.30)	0.64			

HRs and 95% CIs are given per 1 SD increase in biomarker expressed in log_2_ of normalized protein expression units.

*CHF* Chronic heart failure, *CHIT1* Chitotriosidase-1, *CRP* C-reactive protein, *DM* Diabetes mellitus, *eGFR* Estimated glomerular filtration rate, *FABP4* Fatty acid-binding protein 4, *Gal3* Galectin 3, *GDF-15* Growth/differentiation factor 15, *IGFBP-1* Insulin-like growth factor-binding protein 1, *hs-TnT* High sensitivity troponin T, *NT-proBNP* N-terminal prohormone brain natriuretic peptide, *NYHA* New York Heart Association, *PAI-1* Plasminogen activator inhibitor 1, *PON3* Paraoxonase 3, *SBP* Systolic blood pressure, *ST2 protein* Suppressor of tumorigenicity 2, *TR* Transferrin receptor protein 1, *vWF* von Willebrand factor.

*HR and 95% CI is given per 10 mmHg change.

^†^HR and 95% CI is given per doubling.

^‡^Covariates include: SBP, NYHA class III or IV, duration of CHF (years), diabetes mellitus, baseline NT-proBNP, baseline hs-TnT.

Subsequently LASSO-derived Model 3 was compared with a model consisting of repeatedly measured biomarkers selected on the basis of their previously reported involvement in HF including NT-proBNP, hs-TnT, hs-CRP, GDF15, ST2, PAI-1, Gal3 (Model 4, Table 2). The discriminative ability of Model 3 was similar to Model 4 with cvAUCs of both models equal to 0.88. The prospective accuracy of both mvJMs (Model 3 and Model 4) exceeded the prospective accuracy of relative risk models consisting of baseline measurements of the same sets of biomarkers, with cvAUCs of 0.81(0.67; 0.95) for the model containing NT-proBNP, ST2, vWF, FABP4, IGFBP1, PAI-1, TfR, CHIT1, PON3 and of 0.80 (0.65; 0.94) for the model containing NT-proBNP, hs-TnT, hs-CRP, GDF15, ST2, PAI-1, Gal3 (Table S7 for Models, Table S8 for AUCs).

The performance of prediction models that included clinical characteristics was also examined. The variables entered into clinical risk model was based on differences between patients that experienced the PE and those who did not, and consisted of systolic blood pressure, NYHA class, duration of chronic heart failure, diabetes mellitus, baseline NT-proBNP, and baseline hs-TnT (Model 1, Table 2); moreover we evaluated the clinical risk model extended with repeated measurements of standard biomarkers NT-proBNP, hs-TnT, eGFR (Model 2, Table 2) and the clinical risk model extended with the repeatedly measured 9 biomarkers that were selected with LASSO: NT-proBNP, ST2, vWF, FABP4, IGFBP1, PAI-1, TfR, CHIT1 and PON3 (Model 3B, Table S2). The discriminative ability was highest for the LASSO-derived Model 3B with the cvAUCs equal 0.82, 0.82 and 0.87 for Model 1, Model 2 and Model 3B, respectively, as presented in Tables 3 and S6.

**Table 3:** Discriminative ability of Models 1–4.

Models	Model 1	Model 2	Model 3	Model 4
Cross-validated AUC (95% confidence intervals)	0.82* (0.68; 0.96)	0.82^†^ (0.75; 0.89)	0.88^†^ (0.86; 0.90)	0.88^†^ (0.85; 0.90)

*AUC* Area under the curve.

Model 1—Baseline biomarkers and clinical covariates.

Model 2—Serially measured standard biomarkers (NT-proBNP, hs-TnT, eGFR) adjusted for confounders: systolic blood pressure, NYHA class III or IV, duration of chronic heart failure, diabetes mellitus, baseline NT-proBNP, baseline hs-TnT.

Model 3—Serially measured biomarkers selected from the proteomic panel based on penalized regression (LASSO).

Model 4—Serially measured biomarkers selected from the proteomic panel based on previous literature.

*Risk at 24 months after the baseline sample was collected.

^†^Blood sample collection time period of 24 months, and risk for the upcoming 12 months after the collection time.

Besides performing clinical adjustment (Model 3B), other extensions of the LASSO-based Model 3 were also explored: adjustment for age and sex (Model 3A) and addition of repeatedly measured established biomarkers NT-proBNP, hs-TnT, eGFR (Model 3C), presented in Table S5. We observed that neither of these extensions improved the discriminative ability of Model 3. Instead, lower AUC values were observed for these Models compared to Model 3, which may in part be the consequence of inclusion of non-informative variables.

Additionally, to evaluate whether adding biomarkers improves the clinical risk model (Model 1), we calculated the NRI for Model 2 (serially measured established biomarkers NT-proBNP, hs-TnT and eGFR) vs. Model 1, and Model 3B (serially measured biomarkers selected from the proteomic panel based on penalized regression) vs Model 1. We found that both biomarker models improved risk prediction, with greater improvement achieved by the penalization-based 9-biomarker model (bootstrap-based NRI equal to 0.12 [− 0.05; 0.32] for Model 2, and 0.24 [0.06;0.41] for Model 3B).

Results of mvJMs that included both level and slope of the biomarkers in relation to the PE are depicted in Table S9 and show that only for hs-TnT, slope was associated with outcome independent of biomarker level. These models did not present better discriminative ability than models containing only levels of biomarkers (Table S10).

The correlations between all the biomarkers used in the models (measured with Olink as well as hs-TnT, hs-CRP and eGFR) are presented in Table S11. The majority of correlations was weak (rho < 0.5) with a couple of moderate correlations (between 0.5 and 0.7) for GDF15 (with FABP4, Gal3, hs-TnT, NT-proBNP, ST2), NT-proBNP (with hs-TnT and IGFBP1) and Gal3 with FABP4. The correlations between baseline and follow-up measurements of biomarkers were mostly moderate (Table S12). The correlations between duration of CHF and biomarker levels were limited (Table S13).

## Discussion

This is the first study that investigated the association between 92 serially measured protein biomarkers and clinical outcome in a CHF population. Out of 92 potential biomarkers, a model containing 9 proteins, NT-proBNP, ST2, vWF, FABP4, IGFBP1, PAI-1, TfR, CHIT1, and PON3, turned out to predict the occurrence of adverse events with high discriminative ability (cvAUC = 0.88). The discriminative ability of this model was higher compared to a model containing repeated measurements of established biomarkers NT-proBNP, hs-TnT, eGFR and adjusted for baseline clinical predictors, and equal to a model containing a set of biomarkers previously shown to be involved in HF (NT-proBNP, hs-TnT, hs-CRP, GDF15, ST2, PAI-1, Gal3).

The 9-biomarker model was based on the LASSO penalization method with cross-validation, ensuring that its predictive capacity was maximized, and the model was subsequently validated with the use of stratified fivefold cross-validation. Moreover 2 out of these 9 biomarkers were independently associated with the PE when evaluated together in a multivariable joint model; although from a statistical point of view, the p-value is not the deciding criterion in LASSO. The discriminative ability of this model being equal to that of a set of biomarkers previously shown to be involved in HF, illustrates the potential value of relatively ‘novel’ biomarkers, representing several biological mechanisms, for prognostication.

Previous studies on circulating proteins in HF have usually examined one or a few proteins at a time, and have traditionally performed measurements at study baseline only or have left several years between (usually at most two) repeated measurements. Our study design with trimonthly blood draws provided us with blood samples taken on average several weeks before occurrence of an adverse event. This enables us to take into account temporal protein evolution in a unique way. Moreover, by using sophisticated techniques, i.e. LASSO and mvJMs, we are able to derive the most informative proteins from the comprehensive, repeatedly measured panel; and to subsequently use the obtained models for dynamic, individualized prognostication. As we have described earlier, JMs allow for dynamic estimation of prognosis for an individual patient based on his/her temporal biomarker evolution available so far^12^. Because in different patients, the same protein level could have a different meaning depending on whether it is stable or rising; information that is missed when only using measurements at, for example, study baseline, and when using simplified statistical approaches.

Among the proteins included in our model, NT-proBNP is the most investigated protein so far in the context of heart failure^12^. It correlates with echocardiographic measures^20^ and remains the only biomarker with class IA recommendation for diagnostic purposes according to ESC Heart Failure guidelines^6^. However, here we show that the predictive value of repeated NT-proBNP measurements as reported previously^12^ may be further increased when NT-proBNP is measured together with 8 other proteins involved in biological mechanisms that have been implicated in HF, i.e. inflammation and cardiac remodeling (ST2, CHIT1), thrombosis and hemostasis (vWF, PAI-1), the endocrine system (FABP4, IGFBP1), iron homeostasis (TfR), oxidative stress (PON3).

For some of these biomarkers (ST2, vWF, PAI-1, TfR) the predictive value of single measurements in HF has already been reported. Soluble ST2 is a marker that has repeatedly been shown to carry predictive value in HF patients, with recent data indicating that ST2 predicts outcome in chronic HFrEF independent of NT-proBNP and hs-TnT^21^. vWF has been referred to as a marker of endothelial dysfunction^22^. Recent evidence indicates that the acute phase of decompensated HF is linked with an endothelial-driven hypercoagulable phenotype in contrast to the platelet-dependent prothrombotic state in CHF^23^. We captured a rise in plasma vWF prior to exacerbation, for the first time showing that endothelial-related hypercoagulability increases already before clinically evident decompensation. Our observation is consistent with previous studies that revealed its association with functional class and adverse outcome, as well as improved risk prediction when vWF was applied concomitantly with NT-proBNP in HF patients^23,24^. In parallel to prothrombotic state in HF, alterations in fibrynolytic system have also been decribed and prognostic significance of PAI-1 has already been documented by Jug et al. and Winter et al.^4,25^. Soluble TfR was identified as a predictor of poor clinical outcomes in patients with acute HF and a predictor of impaired exercise capacity in patients with HF^3,26^. A very recent publication by Sierpinski et al. identified soluble TfR as the most accurate peripheral blood biomarker of iron deficiency (ID) diagnosed in bone marrow biopsy. Of note, depleted iron stores were observed in 80% of ischemic HFrEF patients. TfR improved outcome prediction beyond established prognosticators, and its additive prognostic value was at least as good or even better than that of plasma NT-proBNP which strongly predicted increased mortality in this group of patients^27^.

For the metabolic biomarkers in our model FABP4 and IGFBP1, much evidence has been collected on their role in cardiac remodelling and HF, but no evident predictive value in HF patients has been demonsated so far. FABP4 is an adipokine released following the activation of beta3 receptors but is also expressed in various cell types including endothelium, immune cells and cardiomyocytes^28–30^. In cardiomyocyte, FABP4 mediates transport of fatty acids from the coronary circulation into the cell^31^ and its knockout induces maladaptive cardiac chamber dilation^28^. On the other hand, it has been reported that FABP4 contributes to cardiac hypertrophy^29^ and contractile dysfunction^32^ as well as reflects the clinical and echocardiographic status of HF patients^33^. The role of IGFBP1, is less understood^34^. However, it was found predictive for incident HF in 3523 Framingham Heart Study participants^35^ and higher levels were observed in HF patients^36^. IGFBP1 is not only involved in insulin growth hormone (IGF) action, but also regulates inhibition or activation of cell growth and induction of apoptosis in an IGF-independent mechanism^37^. As a regulator of IGF availability, it may play a role in multiple hormonal and metabolic deficiency syndrome (MHDS), whose importance for HF patients prognosis has recently been revealed by the investigators of the T.O.S.CA. registry^38^.

For CHIT1 and PON3, less evidence is available. CHIT1 belongs to the family of chitinases, represented in human organism by CHIT1 and acid mammalian chitinase (AMCase). Our knowledge on CHIT1 is relatively new, as its structure was fully described in 2016^39^. It is mainly expressed in innate immune cells such as activated macrophages and neutrophils and has been identified as an early biomarker of atherosclerosis^40^. No reports on CHIT1 in HF exist so far. However, bearing in mind the innate immune system activation in HF^41^, we hypothesize that CHIT1 may be pathophysiologically related to HF and requires further research. PON3 belongs to the paraoxonase multigene family (PON1, PON2, PON3), that has been shown to play an important role in pathogenesis of coronary artery disease^42^, with its role in heart failure being less understood. However in a preclinical study, the paraoxonase gene cluster has been shown to be protective against cardiac hypertrophy, with PON3 becoming upregulated in heart muscle in the course of remodelling^43^. Its prognostic role has not been evaluated so far, with the exception of a cohort of heart transplant recipients with cardiac allograft vasculopathy, in whom decreased PON3 was associated with MACE^27^.

The search for optimal prognostic biomarkers in heart failure is ongoing and the lack of evidence in this area was emphasized in the ESC Heart Failure 2016 guidelines^6,44^. According to the new classification of biomarkers proposed by Salzano et al.^45^, they can be divided into categories based on their potential for application in community-based screening, diagnosis, risk stratification, phenotyping, and management/tailoring treatment. NT-proBNP, the gold standard for diagnostic purposes, has previously shown moderate predictive value with an AUC of 0.672 for the prediction of cardiovascular death and 0.687 for HF hospitalization, which was higher than ST2 (0.622 and 0.626 respectively) but lower than hs-TnT (0.697 and 0.701)^21^. In our cohort, repeated measurements [median of 9 (IQR 5–10) tri-monthly samplings] showed higher AUCs of 0.83 for NT-proBNP and 0.75 for hs-TnT for the composite endpoint, both adjusted for clinical variables. With only 2–3 samples per patient, the LASSO-derived model 3 yielded cvAUC of 0.88. The results of our study underline that repeated measurements of multiple biomarkers reflecting different pathophysiological pathways convey incremental prognostic value to single or even repeated biomarker measurements of established biomarkers.

Although further research is warranted, our results may carry potential for clinical use. With the current advancements in the understanding of the pathophysiology and treatment of HF (e.g. newly approved indication for SGLT2 inhibitors)^46^, the pharmacological armamentarium against HF may further expand in the coming years^47^. Patients may profit from a more personalized diagnostic and prognostic evaluation, as observed in a very recent pre-specified biomarker study from DECLARE-TIMI 58 (a randomized, double-blind, placebo-controlled CV outcomes trial of dapagliflozin) where patients with higher NT-proBNP and hs-TnT derived larger absolute risk reductions with dapagliflozin^48^. Based on our results, we propose to combine information from proteomic panel measurements with advanced statistical methodology, which examines patients’ individual biomarker trajectories that encompass not only assessment of neurohumoral activation, myocardial stretch and damage but also coagulation/fibrinolysis and hormonal/metabolic dysfunction, and may serve as a basis for personalized prognostication. Utilized in this way, repeated biomarker measurements may alter clinical risk estimates and thus have consequences for treatment. Although application of such a dynamic, biomarker-guided strategy still requires investigation in randomized control-trials and validation by methods that yield absolute values of biomarker levels (in contrast to relative units provided by the Olink panel), such as mass spectrometry or immuno-enzymatic methods, we hypothesize that in the long term such a tool may facilitate decision making.

Some limitations of this study warrant consideration. Firstly, our study population was limited in size and focused on HFrEF patients only; therefore, the results should not be extrapolated to HFpEF. Secondly, the Olink panel consists of pre-selected biomarkers, and further studies are warranted that apply wide-scale human proteome identification in HF patients. Thirdly, the Olink panel does not provide absolute concentrations. Therefore, the models containing these proteins should be validated in studies where proteins are measured in absolute concentrations based on validated assays. Finally, although with 66 events in the study population, the 9-biomarker model can be justified in terms of current views on the acceptable number of events per variable, the clinically adjusted 9-biomarker model should be viewed critically.

In conclusion, 9 out of 92 serially measured circulating proteins, related to processes which are dysregulated in cardiovascular disease, provided the optimal multivariable model for occurrence of adverse clinical events in HF patients: NT-proBNP, ST2, vWF, FABP4, IGFBP1, PAI-1, TfR, CHIT1, and PON3. Two proteins were particularly important (NT-proBNP, vWF). While these results should be confirmed by other studies, they suggest that a panel containing these proteins could be useful for personalized risk assessment of patients with HF.

## Supplementary Information

Supplementary Information.
