# Supplementary material for: Dynamic personalized risk prediction in chronic heart failure patients: a longitudinal, clinical investigation of 92 biomarkers (Bio-SHiFT study)
Source: Sci Rep. 2022 Feb 18;12:2795. doi: 10.1038/s41598-022-06698-3 (PMC8857321; doi:10.1038/s41598-022-06698-3)
Supplement: Supplementary file 1 — Supplementary Information. [file 41598_2022_6698_MOESM1_ESM.docx]

**SUPPLEMENTAL MATERIAL**

**Dynamic personalized risk prediction in chronic heart failure patients: a longitudinal, clinical investigation of 92 biomarkers (Bio-SHiFT Study)**

**Short title**: Serial measurements of 92 proteins in CHF patients (Bio-SHiFT)

**Authors**: Dominika Klimczak-Tomaniak MD PhD^a,b^, Marie de Bakker, MSc^a^, Elke Bouwens MD^a^, K. Martijn Akkerhuis MD PhD^a^, Sara Baart PhD^c^, Dimitris Rizopoulos PhD^c^, Henk Mouthaan MSc^d^, Jan van Ramshorst MD PhD^e^, Tjeerd Germans MD PhD^e^, Alina Constantinescu MD PhD^a^, Olivier Manintveld MD PhD^a^, Victor Umans MD PhD^e^, Eric Boersma PhD^a^, Isabella Kardys MD PhD^a^

1. Department of Cardiology, Erasmus MC, University Medical Center Rotterdam, the Netherlands
2. Department of Cardiology, Hypertension and Internal Medicine, Medical University of Warsaw, Warsaw, Poland
3. Department of Biostatistics, Erasmus MC, University Medical Center Rotterdam, the Netherlands
4. Olink Proteomics AB, Uppsala, Sweden
5. Department of Cardiology, Northwest Clinics, Alkmaar, the Netherlands

**Corresponding author:** Isabella Kardys, MD PhD; Erasmus MC, University Medical Center Rotterdam, Department of Cardiology, room NA-316, P.O. Box 2040, 3000 CA Rotterdam, the Netherlands; T +31650032051; Fax number: +31 107044759; E-mail [i.kardys@erasmusmc.nl](mailto:i.kardys@erasmusmc.nl)

**Supplemental figures and tables**

**Figure S1. Inclusion and exclusion criteria.**

**
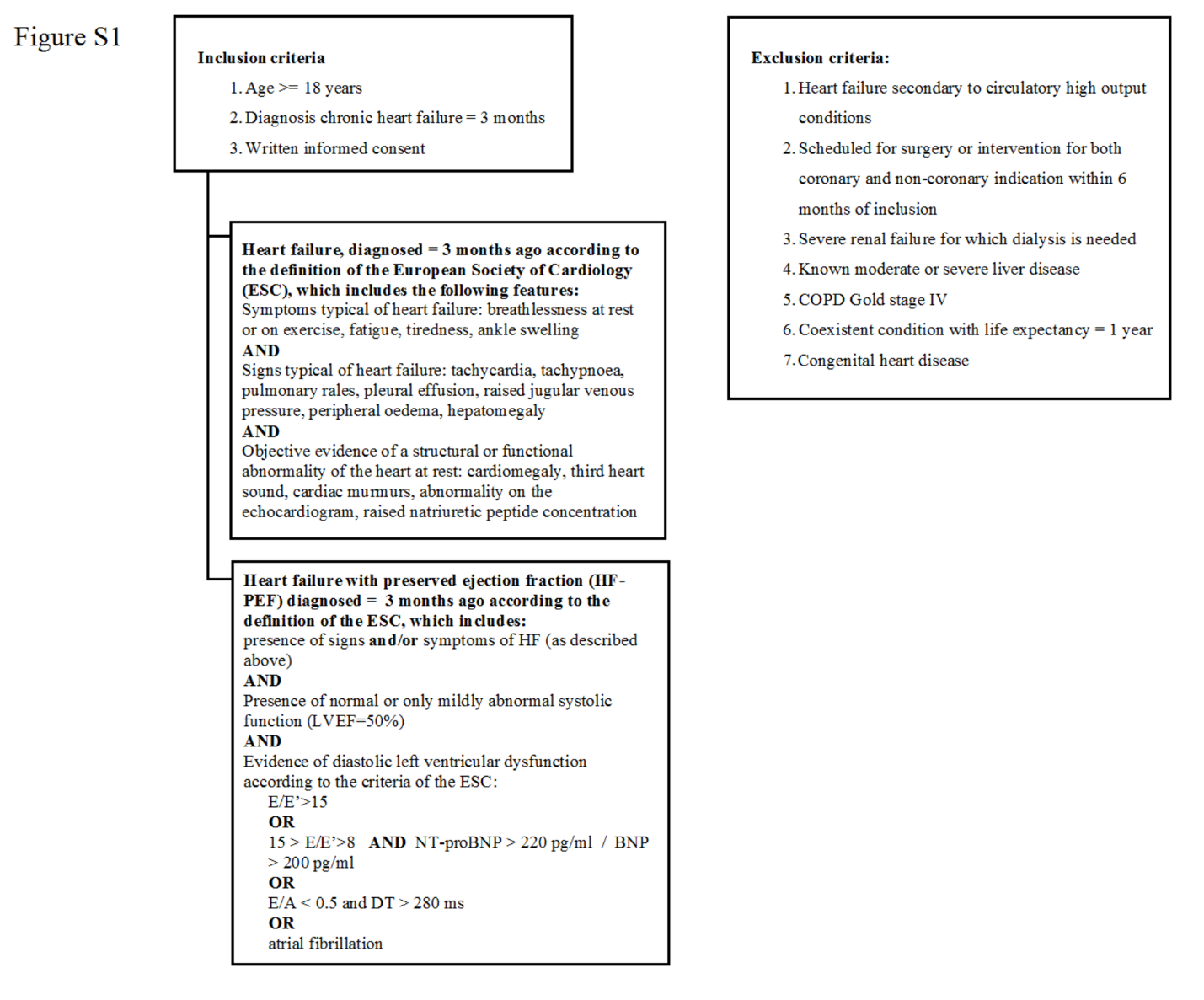
**

**Figure S2. Heatmap of 92 biomarkers measured in baseline blood samples of 250 patients with the primary endpoint and those who remained endpoint-free, clustered unsupervised within the groups.** On the vertical axis, biomarkers are depicted, On the horizontal axis, individual patients are depicted. Protein expression is expressed on a color scale ranging from blue (low) to red (high). Spearman correlation based distance was used as the dissimilarity measure between the standardized biomarkers. The Euclidean distance was used as the dissimilarity measure between patient samples. Ward’s minimum variance method was used as the agglomeration linkage method.


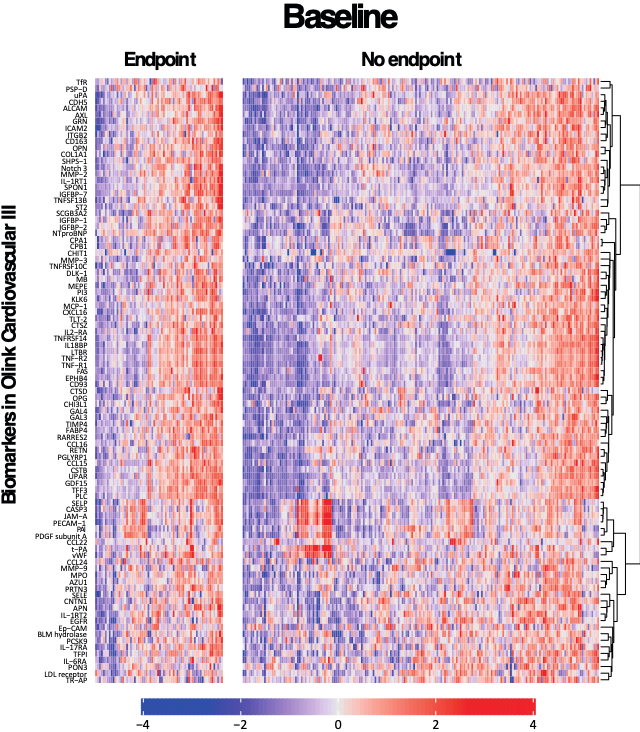


**Figure S3. Heatmap of 92 biomarkers measured in the last available blood samples of 250 patients with the primary endpoint and those who remained endpoint-free, clustered unsupervised within the groups.** On the vertical axis, biomarkers are depicted, On the horizontal axis, individual patients are depicted. Protein expression is expressed on a color scale ranging from blue (low) to red (high). Spearman correlation based distance was used as the dissimilarity measure between the standardized biomarkers. The Euclidean distance was used as the dissimilarity measure between patient samples. Ward’s minimum variance method was used as the agglomeration linkage method.


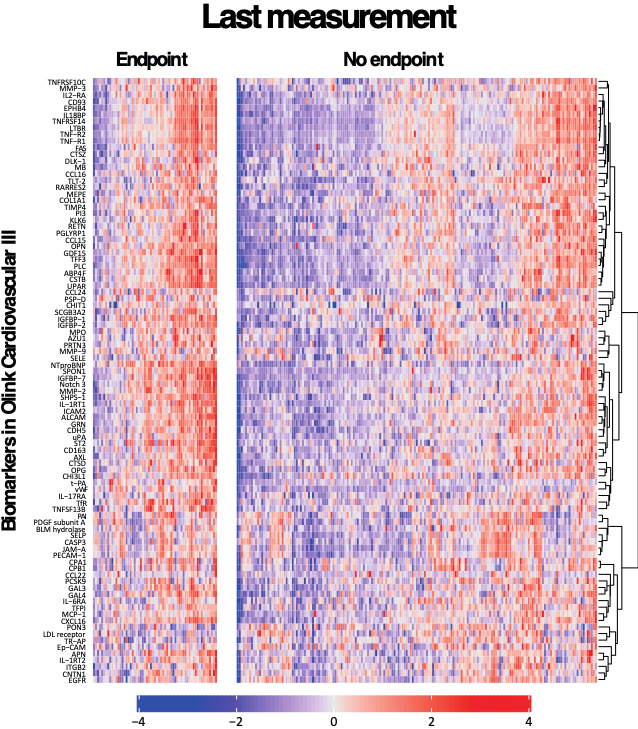


**Fig. S4. Heatmap of 9 biomarkers selected after penalization and introduced into Model 3, measured in baseline blood samples of 250 patients with the primary endpoint and those who remained endpoint-free, clustered unsupervised within the groups.** On the vertical axis, biomarkers are depicted, On the horizontal axis, individual patients are depicted. Protein expression is expressed on a color scale ranging from blue (low) to red (high). Spearman correlation based distance was used as the dissimilarity measure between the standardized biomarkers. The Euclidean distance was used as the dissimilarity measure between patient samples. Ward’s minimum variance method was used as the agglomeration linkage method. Abbreviations: CHIT1, chitotriosidase-1; FABP4, fatty acid-binding protein 4; IGFBP-1, Insulin-like growth factor-binding protein 1; NT-proBNP, N-terminal prohormone brain natriuretic peptide; PAI-1, plasminogen activator inhibitor 1; PON3, paraoxonase 3; ST2 protein, suppressor of tumorigenicity 2; TfR, transferrin receptor protein 1; vWF, von Willebrand factor.


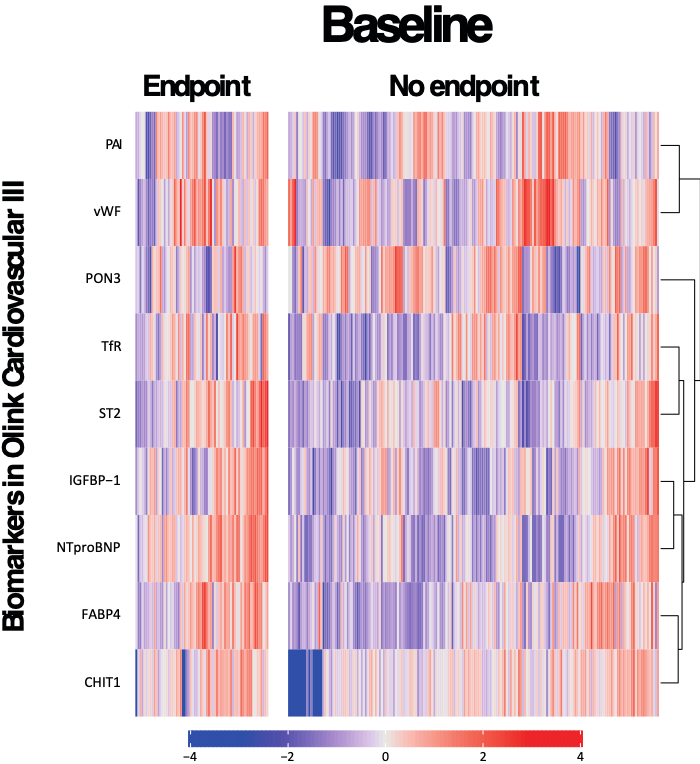


**Fig. S5. Heatmap of 9 biomarkers selected after penalization and introduced into Model 3, measured in the last available blood samples of 250 patients with the primary endpoint and those who remained endpoint-free, clustered unsupervised within the groups.** On the vertical axis, biomarkers are depicted, On the horizontal axis, individual patients are depicted. Protein expression is expressed on a color scale ranging from blue (low) to red (high).Abbreviations: CHIT1, chitotriosidase-1; FABP4, fatty acid-binding protein 4; IGFBP-1, Insulin-like growth factor-binding protein 1; NT-proBNP, N-terminal prohormone brain natriuretic peptide; PAI-1, plasminogen activator inhibitor 1; PON3,paraoxonase 3; ST2 protein, suppressor of tumorigenicity 2; TfR, transferrin receptor protein 1; vWF, von Willebrand factor.


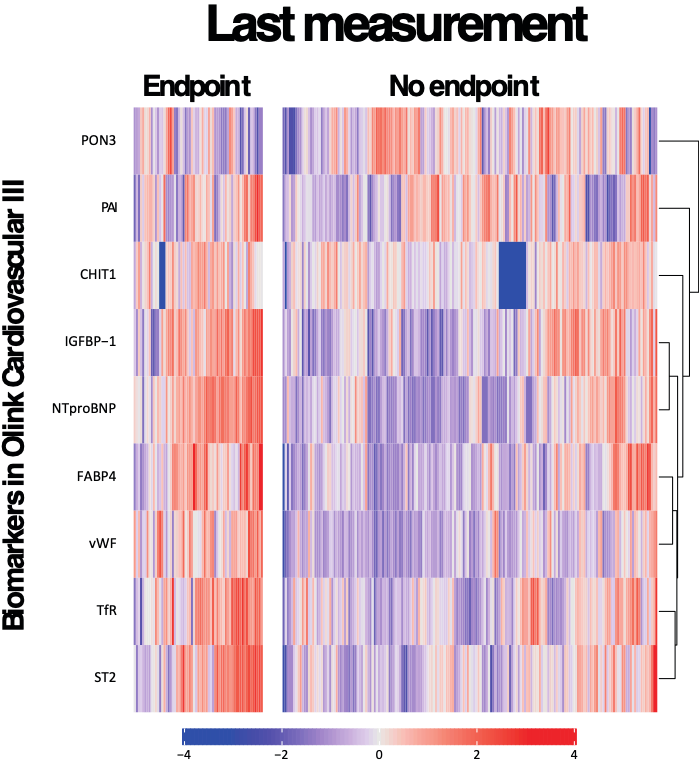


**Table S1. Characteristics of patients (N = 250) stratified according to the occurrence of the primary endpoint (PE).**

| **Variable** | | **PE** | | **No PE** | | **P value** |
| --- | --- | --- | --- | --- | --- | --- |
| **Demographics** | | |  | |  | |
| Age (years) | 71.4 (19.0) | | | 66.2 (15.8) | | 0.043 |
| Male gender | 52 (79%) | | | 132 (72%) | | 0.26 |
| **Clinical characteristics** | | |  | |  | |
| BMI (kg/m2) | | 27.1 (5.71) | | 26.4 (5.88) | | 0.95 |
| Systolic blood pressure (mmHg) | | 11.3 (2.35) | | 12.4 (2.45) | | 0.007* |
| Diastolic blood pressure (mmHg) | | 70 (17) | | 74 (15) | | 0.05 |
| Pulse (beats/min) | | 70 (15) | | 66 (12) | | 0.26 |
| eGFR (ml/min/1.73 m^2^) | | 53.2 (33.2) | | 59.9 (33.9) | | 0.24 |
| **Features of HF** | | |  | |  | |
| LVEF, % | | 28.5 (13) | | 32 (13) | | 0.07 |
| NYHA class III or IV | | 29 (44%) | | 33 (18%) | | <0.001* |
| Duration of HF | | 7.23 9.55 | | 3.77 6.69 | | <0.001* |
| Ischemic aetiology | | 30 (45%) | | 104 (56%) | | 0.12 |
| **Medical history** | | |  | |  | |
| Myocardial infarction | | 32 (48%) | | 63 (34%) | | 0.06 |
| PCI | | 26 (40%) | | 55 (30%) | | 0.15 |
| CABG | | 12 (18%) | | 30 (16%) | | 0.72 |
| Pacemaker | | 12 (20%) | | 28 (16%) | | 0.46 |
| CRT | | 19 (28%) | | 59 (32%) | | 0.73 |
| ICD | | 44 (67%) | | 105 (42%) | | 0.17 |
| AF | | 33 (50%) | | 64 (35%) | | 0.03 |
| Hypertension | | 34 (51%) | | 79 (43%) | | 0.23 |
| Diabetes mellitus | | 29 (44%) | | 48 (26%) | | 0.007* |
| Known hypercholesterolemia | | 29 (44%) | | 65 (36%) | | 0.27 |
| COPD | | 12 (18%) | | 19 (10%) | | 0.09 |
| **Baseline biomarker concentrations** | | |  | |  | |
| NT-proBNP (pmol/l) | | 297 (343) | | 94 (174) | | <0.001* |
| Hs-TnT (ng/l) | | 30.0 (28.1) | | 13.8 (18.7) | | <0.001* |
| CRP (mg/l) | | 2.95 3.87 | | 1.8 3.47 | | 0.02 |

Categorical variables are expressed as count (percentage). Values of continuous variables are expressed as mean ± standard deviation or as median (interquartile range) in case of skewed distribution. * significant with new p threshold = 0.007 after Benjamini-Hochberg procedure with false discovery rate (FDR)=0.05. Abbreviations: CABG, coronary artery bypass grafting; COPD, chronic obstructive pulmonary disease; DBP, diastolic blood pressure; Hs-TnT, high-sensitivity troponin T; LVEF, left ventricular ejection fraction; MI, myocardial infarction; NT-proBNP, N-terminal prohormone of brain natriuretic peptide; PCI, percutaneous coronary intervention; SBP, systolic blood pressure.

**Table S2. Univariable joint models evaluating the association between the 92 individual Olink biomarkers and the PE.**

| **Biomarker** | **HR (95% CI)** | **p** |
| --- | --- | --- |
| **ALCAM** | 2.37 (1.63; 3.49) | <0.001 |
| **AP-N** | 2.13 (1.55; 2.96) | <0.001 |
| **AXL** | 1.62 (1.19; 2.18) | 0.006 |
| **AZU1** | 1.35 (1.08; 1.68) | 0.016 |
| **BLM hydrolase** | 1.05 (0.76; 1.41) | 0.715 |
| **CASP3** | 1.14 (0.80; 1.63) | 0.459 |
| **CCL15** | 1.77 (1.37; 2.29) | <0.001 |
| **CCL16** | 1.67 (1.26; 2.23) | 0.001 |
| **CCL22** | 1.05 (0.81; 1.30) | 0.651 |
| **CCL24** | 1.26 (0.97; 1.65) | 0.082 |
| **CD163** | 2.56 (1.88; 3.71) | <0.001 |
| **CD93** | 2.31 (1.67; 3.29) | <0.001 |
| **CDH5** | 2.11 (1.52; 2.97) | <0.001 |
| **CHI3L1** | 2.11 (1.58; 2.95) | <0.001 |
| **CHIT1** | 5.7 (1.23; 48.39) | 0.002 |
| **CNTN1** | 1.00 (0.70; 1.45) | 1.00 |
| **COL1A1** | 1.90 (1.38; 2.67) | <0.001 |
| **CPA1** | 1.50 (1.10; 2.03) | 0.012 |
| **CPB1** | 1.23 (0.91; 1.64) | 0.175 |
| **CSTB** | 2.48 (1.87; 3.36) | <0.001 |
| **CTSD** | 1.79 (1.38; 2.35) | <0.001 |
| **CTSZ** | 1.32 (1.00; 1.75) | 0.052 |
| **CXCL16** | 2.57 (1.85; 3.72) | <0.001 |
| **DLK1** | 1.28 (0.95; 1.70) | 0.100 |
| **EGFR** | 0.79 (0.56; 1.13) | 0.171 |
| **Ep - CAM** | 0.85 ( 0.64; 1.1) | 0.227 |
| **EPHB4** | 1.90 (1.50; 2.46) | <0.001 |
| **FABP4** | 2.51 (1.90; 3.29) | <0.001 |
| **FAS** | 1.28 (1.01; 1.61) | 0.039 |
| **GAL3** | 1.96 (1.42; 2.73) | <0.001 |
| **GAL4** | 1.89 (1.42; 2.54) | <0.001 |
| **GDF-15** | 3.30 (2.47; 4.53) | <0.001 |
| **GRN** | 2.18 (1.55; 3.13) | <0.001 |
| **ICAM-2** | 2.19 (1.55; 3.20) | <0.001 |
| **IGFBP-1** | 3.64 (2.59; 5.40) | <0.001 |
| **IGFBP-2** | 3.06 (2.17; 4.33) | <0.001 |
| **IGFBP-7** | 2.59 (2.05; 3.32) | <0.001 |
| **IL-17RA** | 1.64 (1.23; 2.15) | 0.001 |
| **IL-1RT1** | 54.28 (7.68; 442.85) | <0.001 |
| **IL-1RT2** | 1.54 (1.15; 2.11) | 0.002 |
| **IL-6RA** | 1.02 (0.78; 1.32) | 0.879 |
| **IL-18BP** | 1.78 (1.33; 2.36) | <0.001 |
| **IL2-RA** | 2.06 (1.48; 2.90) | <0.001 |
| **ITGB2** | 0.99 (0.71; 1.38) | 0.943 |
| **JAM-A** | 1.86 (1.32; 2.71) | 0.001 |
| **KLK6** | 1.6 0 (1.20; 2.12) | 0.003 |
| **LDL receptor** | 0.64 (0.47; 0.87) | 0.008 |
| **LTBR** | 2.17 (1.67; 2.83) | <0.001 |
| **MB** | 1.41 (1.1; 1.81) | 0.007 |
| **MCP-1** | 1.15 (0.94; 1.36) | 0.164 |
| **MEPE** | 1.34 (1.01; 1.79) | 0.044 |
| **MMP-2** | 3.58 (2.39; 5.45) | <0.001 |
| **MMP-3** | 1.58 (1.21; 2.04) | 0.001 |
| **MMP-9** | 2.71 (1.74; 4.66) | <0.001 |
| **MPO** | 1.72 (1.25; 2.41) | <0.001 |
| **Notch 3** | 3.26 (2.29; 4.76) | <0.001 |
| **NT-proBNP** | 5.14 (3.69, 7.05) | <0.001 |
| **OPG** | 2.22 (1.68; 3.01) | <0.001 |
| **OPN** | 2.31 (1.73; 3.22) | <0.001 |
| **PAI** | 1.55 (1.10; 2.18) | 0.004 |
| **PCSK9** | 1.39 (0.97; 1.96) | 0.08 |
| **PDGF subunit A** | 86.8 (16.9; 278.04) | <0.001 |
| **PECAM-1** | 1.40 (0.98; 1.98) | 0.068 |
| **PGLYRP1** | 1.84 (1.41; 2.43 | <0.001 |
| **PI3** | 1.57 (1.22; 2.00) | <0.001 |
| **PLC** | 1.91 (2.61; 3.60) | <0.001 |
| **PON3** | 0.59 (0.45; 0.77) | <0.001 |
| **PRTN3** | 1.57 (1.21; 1.99) | 0.001 |
| **PSP-D** | 1.75 (1.34; 2.26) | <0.001 |
| **RARRES2** | 1.60 (1.20; 2.45) | 0.002 |
| **RETN** | 1.74 (1.34; 2.26) | <0.001 |
| **SCGB3A2** | 1.58 (1.24; 2.01) | <0.001 |
| **SELE** | 1.08 (0.82; 1.39) | 0.541 |
| **SELP** | 1.39 (0.99; 1.96) | 0.056 |
| **SHPS-1** | 2.66 (1.93; 3.78) | <0.001 |
| **SPON1** | 1.65 (1.38; 1.98) | <0.001 |
| **ST2** | 5.31 (3.48; 9.79) | <0.001 |
| **t-PA** | 82.27 (26.39; 242.02) | <0.001 |
| **TFF3** | 2.21 (1.75; 2.82) | <0.001 |
| **TFPI** | 1.36 (0.95; 1.93) | 0.088 |
| **TIMP4** | 2.48 (1.73; 3.62) | <0.001 |
| **TLT-2** | 1.16 (0.89; 1.49) | 0.268 |
| **TNF-R1** | 2.42 (1.85; 3.20) | <0.001 |
| **TNF-R2** | 1.37 (1.15; 1.58) | 0.003 |
| **TNFRSF10C** | 1.57 (1.20; 2.08) | 0.002 |
| **TNFRSF14** | 1.97 (1.53; 2.54) | <0.001 |
| **TNFSF13B** | 17.37 (5.21; 121.02) | <0.001 |
| **TfR** | 10.90 (6.02; 20.55) | <0.001 |
| **TR-AP** | 0.55 (0.41; 0.74) | <0.001 |
| **uPA** | 2.10 (1.53; 2.88) | <0.001 |
| **U-PAR** | 3.52 (2.49; 5.05) | <0.001 |
| **vWF** | 76.21 (19.42; 288.22) | <0.001 |

Abbreviations explained in Text S2 below.

**Table. S3. Multivariable models predicting clinical outcome in the study population based on 2 samples in PE and non-PE patients (N = 250 patients).**

| **Model 2.2**  **Serially measured established biomarkers adjusted for clinical covariates†** | | | **Model 3.2**  **Serially measured biomarkers selected from the proteomic panel based on penalized regression (LASSO)** | | | **Model 4.2**  **Serially measured biomarkers selected from the proteomic panel based on previous literature** | | |
| --- | --- | --- | --- | --- | --- | --- | --- | --- |
| **Variable** | **HR**  **(95% CI)** | **P value** | **Variable** | **HR**  **(95% CI)** | **p value** | **Variable** | **HR**  **(95% CI)** | **P value** |
| **NT-proBNP*** | 2.78  (1.55; 5.65) | <0.001 | **NT-proBNP*** | 1.79  (1.24; 2.75) | 0.002 | **NT-proBNP*** | 1.79  (1.17; 2.77) | 0.007 |
| **hs-TnT†*** | 1.25  (0.49; 3.31) | 0.666 | **ST2** | 0.81  (0.27; 1.90) | 0.667 | **hs-TnT*** | 1.26  (0.75; 2.07) | 0.364 |
| **eGFR†*** | 2.81  (0.93; 11.47) | 0.073 | **vWF** | 3.89  (1.02; 28.09) | 0.046 | **hsCRP*** | 1.36  (0.94; 2.01) | 0.101 |
|  |  |  | **FABP4** | 0.95  (0.26; 2.48) | 0.997 | **GDF-15** | 0.97  (0.30; 2.76) | 0.997 |
|  |  |  | **IGFBP1** | 1.19  (0.51; 3.21) | 0.720 | **ST2** | 1.13  (0.56; 2.28) | 0.719 |
|  |  |  | **PAI-1** | 0.95  (0.35; 2.44) | 0.933 | **PAI-1** | 1.31  (0.69; 2.50) | 0.399 |
|  |  |  | **TfR** | 1.79  (0.76; 5.18) | 0.191 | **Gal3** | 0.97  (0.44; 2.24) | 0.939 |
|  |  |  | **CHIT1** | 0.82  (0.49; 1.38) | 0.418 |  |  |  |
|  |  |  | **PON3** | 0.83  (0.46; 1.46) | 0.534 |  |  |  |

HRs and 95% CIs are given per 1 SD increase in biomarker expressed in log_2_ of normalized protein expression units; *HR and 95% CI is given per doubling; †covariates include: SBP, NYHA class III or IV, duration of CHF (years), diabetes mellitus, baseline NT-proBNP, baseline hs-TnT. Abbreviations: CHF, chronic heart failure; CHIT1, chitotriosidase-1; CRP, C-reactive protein; DM, diabetes mellitus; eGFR, estimated glomerular filtration rate; FABP4, fatty acid-binding protein 4; Gal3, Galectin 3; GDF-15, Growth/differentiation factor 15; IGFBP-1, Insulin-like growth factor-binding protein 1; hs-TnT, high sensitivity troponin T; NT-proBNP, N-terminal prohormone brain natriuretic peptide; NYHA, New York Heart Association; PAI-1, plasminogen activator inhibitor 1; PE, primary endpoint; PON3, paraoxonase 3; SBP, systolic blood pressure, ST2 protein, suppressor of tumorigenicity 2; TR, transferrin receptor protein 1; vWF, von Willebrand factor.

**Table S4. Discriminative ability of Models 2.2 - 4.2.**

| **Models** | **Model 2.2** | **Model 3.2** | **Model 4.2** |
| --- | --- | --- | --- |
| **Cross-validated AUC (95% confidence intervals)*** | 0.85  (0.80; 0.90) | 0.82  (0.74; 0.89) | 0.82  (0.77; 0.86) |

Model 2.2 - Serially measured standard biomarkers (NT-proBNP, hs-TnT, eGFR) adjusted for confounders: systolic blood pressure, NYHA class III or IV, duration of chronic heart failure, diabetes mellitus, baseline NT-proBNP, baseline hs-TnT.

Model 3.2 - Serially measured biomarkers selected from the proteomic panel based on penalized regression (LASSO)

Model 4.2 - Serially measured biomarkers selected from the proteomic panel based on previous literature

* blood sample collection time period of 24 months, and risk for the upcoming 12 months after the collection time

Abbreviations: AUC, area under the curve

**Table S5. Extensions of Model 3.**

| **Model 3**  **Serially measured biomarkers selected from the proteomic panel based on penalized regression (LASSO)** | | | **Model 3A**  **Model 3 adjusted for age and sex** | | | **Model 3B**  **Model 3 adjusted for clinical covariates*** | | | **Model 3C**  **Serially measured hsTnT† and eGFR incorporated into Model 3** | | |
| --- | --- | --- | --- | --- | --- | --- | --- | --- | --- | --- | --- |
| **Variable** | **HR**  **(95% CI)** | **p value** | **Variable** | **HR**  **(95% CI)** | **p**  **value** | **Variable** | **HR**  **(95% CI)** | **p value** | **Variable** | **HR**  **(95% CI)** | **p**  **value** |
| **NT-proBNP** | 1.79  (1.35; 2.45) | <0.001 | **NT-proBNP** | 1.70  (1.32; 2.17) | <0.001 | **NT-proBNP** | 2.18  (1.60; 3.08) | <0.001 | **NT-proBNP** | 1.72  (1.26; 2.29) | <0.001 |
| **ST2** | 0.78  (0.45; 1.42) | 0.39 | **ST2** | 0.94  (0.53; 1.65) | 0.69 | **ST2** | 0.93  (0.61; 1.39) | 0.59 | **ST2** | 0.74  (0.41; 1.23) | 0.25 |
| **vWF** | 3.21  (1.23; 9.44) | 0.02 | **vWF** | 2.34  (1.09; 5.35) | 0.03 | **vWF** | 2.16  (1.19; 3.99) | 0.02 | **vWF** | 3.21  (1.34; 7.59) | 0.01 |
| **FABP4** | 1.06  (0.68; 1.64) | 0.78 | **FABP4** | 1.18  (0.74; 1.82) | 0.41 | **FABP4** | 0.99  (0.71; 1.40) | 0.79 | **FABP4** | 1.32  (0.73; 2.41) | 0.32 |
| **IGFBP1** | 1.23  (0.68; 2.52) | 0.54 | **IGFBP1** | 1.23  (0.75; 2.11) | 0.40 | **IGFBP1** | 1.15  (0.75; 1.77) | 0.44 | **IGFBP1** | 1.14  (0.67; 2.01) | 0.61 |
| **PAI-1** | 1.11  (0.65; 1.85) | 0.69 | **PAI-1** | 1.35  (0.86; 2.20) | 0.19 | **PAI-1** | 1.28  (0.87; 1.87) | 0.18 | **PAI-1** | 0.97  (0.55; 1.75) | 0.89 |
| **TfR** | 1.37  (0.85; 2.25) | 0.20 | **TfR** | 1.49  (0.99; 2.27) | 0.05 | **TfR** | 1.18  (0.85; 1.63) | 0.26 | **TfR** | 1.36  (0.86; 2.20) | 0.17 |
| **CHIT1** | 0.84  (0.59; 1.20) | 0.30 | **CHIT1** | 0.79  (0.61; 1.06) | 0.11 | **CHIT1** | 0.77  (0.59; 1.00) | 0.05 | **CHIT1** | 0.88  (0.65; 1.22) | 0.38 |
| **PON3** | 0.92  (0.65; 1.30) | 0.64 | **PON3** | 0.99  (0.70; 1.44) | 0.81 | **PON3** | 1.00  (0.73; 1.37) | 0.80 | **PON3** | 1.00  (0.69; 1.47) | 0.95 |
|  |  |  |  |  |  |  |  |  | **hs-TnT**† | 1.19  (0.85; 1.65) | 0.29 |
|  |  |  |  |  |  |  |  |  | **eGFR**† | 2.21  (0.74; 7.20) | 0.15 |

HRs and 95% CIs are given per 1 SD increase in biomarker expressed in log_2_ of normalized protein expression units. * covariates include: systolic blood pressure, NYHA class III or IV, duration of CHF (years), diabetes mellitus, baseline NT-proBNP, baseline hsTnT; † HR and 95% CI is given per doubling; Abbreviations: CHF, chronic heart failure; CHIT1, chitotriosidase-1; CRP, C-reactive protein; DM, diabetes mellitus; eGFR, estimated glomerular filtration rate; FABP4, fatty acid-binding protein 4; Gal3, Galectin 3; GDF-15, Growth/differentiation factor 15; IGFBP-1, Insulin-like growth factor-binding protein 1; hsTnT, high sensitivity troponin T; NTproBNP, N-terminal prohormone brain natriuretic peptide; NYHA, New York Heart Association; PAI-1, plasminogen activator inhibitor 1; PON3, paraoxonase 3; SBP, systolic blood pressure, ST2 protein, suppressor of tumorigenicity 2; TfR, transferrin receptor protein 1; vWF, von Willebrand factor.

**Table S6.** **Discriminative ability of Model 3 and its extensions.**

| **Models** | **Model 3** | **Model 3A** | **Model 3B** | **Model 3C** |
| --- | --- | --- | --- | --- |
| **Cross-validated ACUs (95% CI)*** | 0.88 (0.86; 0.90) | 0.87 (0.84; 0.89) | 0.88 (0.81; 0.94) | 0.89 (0.86; 0.91) |

Model 3 **-** Serially measured biomarkers selected from the proteomic panel based on penalized regression (LASSO)

Model 3A **-** Model 3 adjusted for age and sex

Model 3B - Model 3 adjusted for clinical covariates: systolic blood pressure, NYHA class III or IV, duration of CHF (years), diabetes mellitus, baseline NT-proBNP, baseline hsTnT

Model 3C - Serially measured hsTnT and eGFR incorporated into Model 3

*blood sample collection time period of 24 months, and risk for the upcoming 12 months after the collection time

Abbreviations: AUC, area under the curve; CI, confidence interval.

**Table S7. Relative risk models containing baseline measurements of biomarkers, with no further adjustments.**

| **LASSO-derived**  **selection** | | | **Previous literature-based selection** | | |
| --- | --- | --- | --- | --- | --- |
| **Variable** | **HR**  **(95% CI)** | **p value** | **Variable** | **HR**  **(95% CI)** | **p**  **value** |
| **NT-proBNP** | 1.48 (1.21, 1.81) | <0.001 | **NT-proBNP** | 1.40 (1.18, 1.68) | <0.001 |
| **ST2** | 1.17 (0.87, 1.58) | 0.30 | **hsTnT** | 1.16 (1.00, 1.36) | 0.05 |
| **vWF** | 1.07 (0.79, 1.45) | 0.64 | **hsCRP** | 1.15 (0.91, 1.46) | 0.25 |
| **FABP4** | 1.22 (0.91, 1.64) | 0.17 | **GDF-15** | 1.55 (1.08, 2.22) | 0.02 |
| **IGFBP1** | 1.19 (0.87, 1.63) | 0.27 | **ST2** | 1.15 (0.87, 0.85) | 0.35 |
| **PAI-1** | 1.03 (0.77, 1.38) | 0.85 | **PAI-1** | 1.14 (0.88, 0.86) | 0.36 |
| **TfR** | 1.14 (0.85, 1.51) | 0.38 | **Gal3** | 0.93 (0.70, 1.25) | 0.65 |
| **CHIT1** | 0.95 (0.72, 1.25) | 0.70 |  |  |  |
| **PON3** | 0.72 (0.58, 0.91) | 0.006 |  |  |  |

Abbreviations: CHIT1, chitotriosidase-1; CRP, C-reactive protein; eGFR, estimated glomerular filtration rate; FABP4, fatty acid-binding protein 4; Gal3, Galectin 3; GDF-15, Growth/differentiation factor 15; IGFBP-1, Insulin-like growth factor-binding protein 1; hsTnT, high sensitivity troponin T; NTproBNP, N-terminal prohormone brain natriuretic peptide; PAI-1, plasminogen activator inhibitor 1; PON3, paraoxonase 3; ST2 protein, suppressor of tumorigenicity 2; TfR, transferrin receptor protein 1; vWF, von Willebrand factor.

| **Models** | **LASSO- derived**  **selection** | **Pathophysiologically-based selection** |
| --- | --- | --- |
| **cvAUC (95% confidence intervals)*** | 0.81 (0.67; 0.95) | 0.80 (0.65; 0.94) |

**Table S8. Discriminative ability of relative risk models presented in Table S7.**

* risk at 24 months after the baseline sample was collected

Abbreviations: cvAUC, cross-validated area under the curve; CI, confidence intervals

**Table S9. Multivariable models containing both biomarker level and slope, in relation to clinical outcome (N = 250 patients).**

| **Model 5**  **Serially measured established biomarkers adjusted for clinical covariates‡** | | | **Model 6**  **Serially measured biomarkers selected from the proteomic panel based on penalized regression (LASSO)** | | | **Model 7**  **Serially measured biomarkers selected from the proteomic panel based on previous literature** | | |
| --- | --- | --- | --- | --- | --- | --- | --- | --- |
| **Variable** | **HR**  **(95% CI)** | **p**  **value** | **Variable** | **HR****  **(95% CI)** | **p value** | **Variable** | **HR**  **(95% CI)** | **p**  **value** |
| **NT-proBNP*** | 1.00  (0.97; 1.04) | 0.50 | **NT-proBNP*** | 1.01  (0.72; 1.42) | 0.95 | **NT-proBNP*** | 1.14  (0.77; 1.62) | 0.44 |
| **hs-TnT*** | 0.95  (0.90; 0.99) | 0.03 | **ST2** | 0.96  (0.45; 2.01) | 0.94 | **hs-TnT*** | 0.71  (0.37; 1.35) | 0.32 |
| **eGFR*** | 0.90 0.73 1.13 | 0.24 | **vWF** | 1.48  (0.97; 2.25) | 0.08 | **hsCRP*** | 1.08  (0.72; 1.54) | 0.57 |
|  |  |  | **FABP4** | 1.00  (0.45; 2.10) | 0.98 | **GDF-15** | 0.79  (0.34; 1.81) | 0.57 |
|  |  |  | **IGFBP1** | 1.00  (0.49; 1.93) | 0.99 | **ST2** | 0.82  (0.37; 1.77) | 0.60 |
|  |  |  | **PAI-1** | 0.83  (0.48; 1.48) | 0.52 | **PAI-1** | 1.05  (0.97; 1.13) | 0.85 |
|  |  |  | **TfR** | 0.89 (0.43; 1.80) | 0.74 | **Gal3** | 1.52  (0.84; 2.76) | 0.15 |
|  |  |  | **CHIT1** | 0.89 (0.27; 2.86) | 0.83 |  |  |  |
|  |  |  | **PON3** | 1.16 (0.72; 2.00) | 0.58 |  |  |  |

*HR per doubling of slope, **HR per 0.1 SD change in slope of Olink panel proteins. ‡ covariates include: SBP, NYHA class III or IV, duration of CHF (years), diabetes mellitus, baseline NT-proBNP, baseline hs-TnT. Abbreviations: 95%CI, 95% confidence intervals; CHF, chronic heart failure; CHIT1, chitotriosidase-1; CRP, C-reactive protein; DM, diabetes mellitus; eGFR, estimated glomerular filtration rate; FABP4, fatty acid-binding protein 4; Gal3, Galectin 3; GDF-15, Growth/differentiation factor 15; IGFBP-1, Insulin-like growth factor-binding protein 1; HR, hazard ratio; hs-TnT, high sensitivity troponin T; NT-proBNP, N-terminal prohormone brain natriuretic peptide; NYHA, New York Heart Association; PAI-1, plasminogen activator inhibitor 1; PON3, paraoxonase 3; SBP, systolic blood pressure, ST2 protein, suppressor of tumorigenicity 2; TR, transferrin receptor protein 1; vWF, von Willebrand factor.

**Table S10. Predictive ability of Models 5-7.**

| **Models** | **Model 5** | **Model 6** | **Model 7** |
| --- | --- | --- | --- |
| **Cross-validated AUC (95% confidence intervals)*** | 0.64  (0.43; 0.86) | 0.85  (0.84; 0.87) | 0.87  (0.85; 0.89) |

Model 5 - Serially measured standard biomarkers (NT-proBNP, hs-TnT, eGFR) adjusted for confounders: systolic blood pressure, NYHA class III or IV, duration of chronic heart failure, diabetes mellitus, baseline NT-proBNP, baseline hs-TnT.

Model 6 - Serially measured biomarkers selected from the proteomic panel based on penalized regression (LASSO)

Model 7 - Serially measured biomarkers selected from the proteomic panel based on previous literature

*blood sample collection time period of 24 months, and risk for the upcoming 12 months after the collection time

Abbreviations: AUC, area under the curve.

**Table S11. Correlations between the baseline values of biomarkers (presented as rho values).**

|  | **CHIT1** | **eGFR** | **FABP4** | **GAL3** | **GDF15** | **hsCRP** | **hs-TnT** | **IGFBP1** | **NT-proBNP** | **PAI1** | **PON3** | **ST2** | **TfR** | **vWF** |
| --- | --- | --- | --- | --- | --- | --- | --- | --- | --- | --- | --- | --- | --- | --- |
| **CHIT1** | 1.00 | -0.26* | 0.39* | 0.31* | 0.45* | 0.10 | 0.40* | 0.35* | 0.33* | -0.12 | -0.05 | 0.22 | 0.22 | 0.04 |
| **eGFR** | -0.26* | 1.00 | -0.44* | -0.2* | -0.39* | -0.02 | -0.24* | -0.16* | -0.27* | 0.25* | -0.02 | -0.12 | -0.09 | -0.04 |
| **FABP4** | 0.39* | -0.44* | 1.00 | 0.61* | 0.68* | 0.37* | 0.43* | 0.25* | 0.39* | 0.12 | -0.11 | 0.36* | 0.33* | 0.19* |
| **GAL3** | 0.31* | -0.20* | 0.61* | 1.00 | 0.51* | 0.24* | 0.29* | 0.28* | 0.30* | 0.12 | 0.18* | 0.35* | 0.30* | 0.17* |
| **GDF15** | 0.45* | -0.39* | 0.68* | 0.51* | 1.00 | 0.30* | 0.61* | 0.45* | 0.58* | -0.07 | -0.16* | 0.50* | 0.39* | 0.22 |
| **hsCRP** | 0.10 | -0.02 | 0.37* | 0.24* | 0.30* | 1.00 | 0.09 | 0.09 | 0.16* | 0.28* | -0.22* | 0.23* | 0.24* | 0.29* |
| **hs-TnT** | 0.40* | -0.24* | 0.43* | 0.29* | 0.61* | 0.09 | 1.00 | 0.39* | 0.62* | -0.16* | -0.2* | 0.29* | 0.25* | 0.06 |
| **IGFBP-1** | 0.35* | -0.16* | 0.25* | 0.28* | 0.45* | 0.09 | 0.39* | 1.00 | 0.51* | -0.18* | 0.11 | 0.33* | 0.16* | 0.06 |
| **NT-proBNP** | 0.33* | -0.27* | 0.39* | 0.3* | 0.58* | 0.16* | 0.62* | 0.51* | 1.00 | -0.22* | -0.06 | 0.37* | 0.28* | 0.06 |
| **PAI1** | -0.12 | 0.25* | 0.12 | 0.12 | -0.07 | 0.28* | -0.16* | -0.18 | -0.22* | 1.00 | -0.11 | 0.17* | 0 | 0.34* |
| **PON3** | -0.05 | -0.02 | -0.11 | 0.18* | -0.16* | -0.22* | -0.20* | 0.11 | -0.06 | -0.11* | 1.00 | 0.01 | -0.05 | -0.19* |
| **ST2** | 0.22 | -0.12 | 0.36* | 0.35* | 0.50* | 0.23* | 0.29* | 0.33* | 0.37* | 0.17 | 0.01 | 1.00 | 0.36* | 0.16* |
| **TfR** | 0.22 | -0.09 | 0.33* | 0.30* | 0.39* | 0.24* | 0.25* | 0.16* | 0.28* | 0.00 | -0.05 | 0.36* | 1.00 | -0.01 |
| **vWF** | 0.04 | -0.04 | 0.19* | 0.17* | 0.22* | 0.29* | 0.06 | 0.06 | 0.06 | 0.34* | -0.19* | 0.16* | -0.01 | 1.00 |

* significant with new p threshold = 0.013 after Benjamini-Hochberg procedure with false discovery rate (FDR)=0.05. Abbreviations: CHIT1, chitotriosidase-1; FABP4, fatty acid-binding protein 4; Gal3, Galectin 3; GDF-15, Growth/differentiation factor 15; hs-CRP, C-reactive protein; hs-TnT, high sensitivity troponin T; IGFBP-1, Insulin-like growth factor-binding protein 1; NTproBNP, N-terminal prohormone brain natriuretic peptide; PAI-1, plasminogen activator inhibitor 1; PON3, paraoxonase 3; ST2 protein, suppressor of tumorigenicity 2; TfR, transferrin receptor protein 1; vWF, von Willebrand factor

**Table S12. Correlations between serial measurements of the biomarkers.**

| **CHIT1** | **1st measurement** | **2nd measurement** | **3rd measurement** |
| --- | --- | --- | --- |
| **1st measurement** | 1.00 | 0.89* | 0.75* |
| **2nd measurement** | 0.89* | 1.00 | 0.80* |
| **3rd measurement** | 0.75* | 0.80* | 1.00 |
| **FABP4** |  |  |  |
| **1st measurement** | 1.00 | 0.78* | 0.77* |
| **2nd measurement** | 0.78* | 1.00 | 0.80* |
| **3rd measurement** | 0.77* | 0.80* | 1.00 |
| **Gal3** |  |  |  |
| **1st measurement** | 1.00 | 0.56* | 0.54* |
| **2nd measurement** | 0.56* | 1.00 | 0.49* |
| **3rd measurement** | 0.54* | 0.49* | 1.00 |
| **GDF15** | |  |  |
| **1st measurement** | 1.00 | 0.81* | 0.54* |
| **2nd measurement** | 0.81* | 1.00 | 0.73* |
| **3rd measurement** | 0.54* | 0.73* | 1.00 |
| **IGFBP1** | |  |  |
| **1st measurement** | 1.00 | 0.69* | 0.43* |
| **2nd measurement** | 0.69* | 1.00 | 0.44* |
| **3rd measurement** | 0.43* | 0.44* | 1.00 |
| **NT-proBNP** | |  |  |
| **1st measurement** | 1.00 | 0.86* | 0.63* |
| **2nd measurement** | 0.86* | 1.00 | 0.77* |
| **3rd measurement** | 0.63* | 0.77* | 1.00 |
| **PAI-1** |  |  |  |
| **1st measurement** | 1.00 | 0.56* | 0.63* |
| **2nd measurement** | 0.56* | 1.00 | 0.55* |
| **3rd measurement** | 0.63* | 0.55* | 1.00 |
| **PON3** |  |  |  |
| **1st measurement** | 1.00 | 0.63* | 0.60* |
| **2nd measurement** | 0.63* | 1.00 | 0.65* |
| **3rd measurement** | 0.60* | 0.65* | 1.00 |
| **ST2** |  |  |  |
| **1st measurement** | 1.00 | 0.70* | 0.55* |
| **2nd measurement** | 0.70* | 1.00 | 0.62* |
| **3rd measurement** | 0.55* | 0.62* | 1.00 |
| **TfR** |  |  |  |
| **1st measurement** | 1.00 | 0.71* | 0.72* |
| **2nd measurement** | 0.71* | 1.00 | 0.74* |
| **3rd measurement** | 0.72* | 0.74* | 1.00 |
| **vWF** |  |  |  |
| **1st measurement** | 1.00 | 0.32* | 0.19 |
| **2nd measurement** | 0.32* | 1.00 | 0.14 |
| **3rd measurement** | 0.19 | 0.14 | 1.00 |

* significant with new p threshold = 0.002 after after Benjamini-Hochberg procedure with false discovery rate (FDR)=0.05. Abbreviations: CHF, chronic heart failure; CHIT1, chitotriosidase-1; eGFR, estimated glomerular filtration rate; FABP4, fatty acid-binding protein 4; Gal3, Galectin 3; GDF-15, Growth/differentiation factor 15; IGFBP-1, Insulin-like growth factor-binding protein 1; hs-CRP, C-reactive protein; hsTnT, high sensitivity troponin T; NTproBNP, N-terminal prohormone brain natriuretic peptide; NYHA, New York Heart Association; PAI-1, plasminogen activator inhibitor 1; PON3, paraoxonase 3; SBP, systolic blood pressure, ST2 protein, suppressor of tumorigenicity 2; TfR, transferrin receptor protein 1; vWF, von Willebrand factor.

**Table S13. Correlations between the duration of CHF and values of Olink biomarker samples at 1^st^, 2^nd^ and 3^rd^ sampling time.**

| **Biomarker** | **rho** | **p value** |
| --- | --- | --- |
| **CHIT1 1st** | 0.12 | 0.058 |
| **CHIT1 2nd** | 0.10 | 0.112 |
| **CHIT1 3rd** | 0.21 | 0.155 |
| **FABP4 1st** | 0.20 | 0.002 |
| **FABP4 2nd** | 0.16 | 0.014 |
| **FABP4 3rd** | 0.12 | 0.431 |
| **GAL3 1st** | 0.16 | 0.013 |
| **GAL3 2nd** | 0.08 | 0.223 |
| **GAL3 3rd** | -0.01 | 0.949 |
| **GDF15 1st** | 0.16 | 0.009 |
| **GDF15 2nd** | 0.19 | 0.003 |
| **GDF15 3rd** | 0.20 | 0.173 |
| **IGFBP.1 1st** | 0.04 | 0.509 |
| **IGFBP.1 2nd** | 0.11 | 0.085 |
| **IGFBP.1 3rd** | 0.02 | 0.886 |
| **NTproBNP 1st** | 0.14 | 0.023 |
| **NTproBNP 2nd** | 0.22 | 0.001 |
| **NTproBNP 3rd** | 0.15 | 0.293 |
| **PAI 1st** | 0.24 | 0.000 |
| **PAI 2nd** | 0.23 | 0.000 |
| **PAI 3rd** | -0.07 | 0.633 |
| **PON3 1st** | -0.10 | 0.129 |
| **PON3 2nd** | -0.15 | 0.019 |
| **PON3 3rd** | -0.01 | 0.947 |
| **ST2 1st** | 0.30 | 0.000 |
| **ST2 2nd** | 0.27 | 0.000 |
| **ST2 3rd** | 0.17 | 0.242 |
| **TR 1st** | 0.15 | 0.019 |
| **TR 2nd** | 0.10 | 0.119 |
| **TR 3rd** | -0.06 | 0.695 |
| **vWF 1st** | 0.14 | 0.030 |
| **vWF 2nd** | 0.13 | 0.042 |
| **vWF 3rd** | 0.00 | 0.992 |

Abbreviations: CHIT1, chitotriosidase-1; FABP4, fatty acid-binding protein 4; Gal3, Galectin 3; GDF-15, Growth/differentiation factor 15; IGFBP-1, Insulin-like growth factor-binding protein 1; NTproBNP, N-terminal prohormone brain natriuretic peptide; PAI-1, plasminogen activator inhibitor 1; PON3, paraoxonase 3; ST2 protein, suppressor of tumorigenicity 2; TfR, transferrin receptor protein 1; vWF, von Willebrand factor.

# Supplemental methods

**Text S1. Definitions used for endpoints**

We used the International Classification of Disease-10^th^ revision (ICD-10), from the World Health Organization, to assign the fatal endpoints [49]. Cardiac death was defined as death from MI or other ischemic heart disease (ICD-10: codes I20-I25), death from other heart disease including HF (codes I30-I45 and I47-I52), sudden cardiac death (code I46), sudden death undefined (code R96) or unwitnessed or ill-described death (codes R98, R99). Hospitalization for acute or worsened HF was defined as a hospitalization for an exacerbation of HF symptoms, in combination with two of the following: BNP or NT-proBNP > 3x upper limit of normal, signs of worsening HF, such as pulmonary rales, raised jugular venous pressure or peripheral oedema, increased dose or intravenous administration of diuretics, or administration of positive inotropic agents [50].

**Text S2. Biomarkers in Olink Cardiovascular III**

Aminopeptidase N (AP-N)

Azurocidin (AZU1)

Bleomycin hydrolase (BLM hydrolase)

C-C motif chemokine 15 (CCL15)

C-C motif chemokine 16 (CCL16)

C-C motif chemokine 22 (CCL22)

C-C motif chemokine 24 (CCL24)

C-X-C motif chemokine 16 (CXCL16)

Cadherin-5 (CDH5)

Carboxypeptidase A1 (CPA1)

Carboxypeptidase B (CPB1)

Caspase-3 (CASP-3)

Cathepsin D (CTSD)

Cathepsin Z (CTSZ)

CD166 antigen (ALCAM)

Chitinase-3-like protein 1 (CHI3L1)

Chitotriosidase-1 (CHIT1)

Collagen alpha-1(I) chain (COL1A1)

Complement component C1q receptor (CD93)

Contactin-1 (CNTN1)

Cystatin-B (CSTB)

E-selectin (SELE)

Elafin (PI3)

Ephrin type-B receptor 4 (EPHB4)

Epidermal growth factor receptor (EGFR )

Epithelial cell adhesion molecule (Ep-CAM)

Fatty acid-binding protein, adipocyte (FABP4)

Galectin-3 (Gal-3)

Galectin-4 (Gal-4)

Granulins (GRN)

Growth/differentiation factor 15 (GDF-15)

Insulin-like growth factor-binding protein 1 (IGFBP-1)

Insulin-like growth factor-binding protein 2 (IGFBP-2)

Insulin-like growth factor-binding protein 7 (IGFBP-7)

Integrin beta-2 (ITGB2)

Intercellular adhesion molecule 2 (ICAM-2)

Interleukin-1 receptor type 1 (IL-1RT1)

Interleukin-1 receptor type 2 (IL-1RT2)

Interleukin-17 receptor A (IL-17RA)

Interleukin-18-binding protein (IL-18BP)

Interleukin-2 receptor subunit alpha (IL2-RA)

Interleukin-6 receptor subunit alpha (IL-6RA)

Junctional adhesion molecule A (JAM-A)

Kallikrein-6 (KLK6 )

Low-density lipoprotein receptor (LDL receptor)

Lymphotoxin-beta receptor (LTBR)

Matrix extracellular phosphoglycoprotein (MEPE)

Matrix metalloproteinase-2 (MMP-2)

Matrix metalloproteinase-3 (MMP-3)

Matrix metalloproteinase-9 (MMP-9)

Metalloproteinase inhibitor 4 (TIMP4)

Monocyte chemotactic protein 1 (MCP-1)

Myeloblastin (PRTN3)

Myeloperoxidase (MPO)

Myoglobin (MB)

N-terminal prohormone brain natriuretic peptide (NT-proBNP)

Neurogenic locus notch homolog protein 3 (Notch 3)

Osteopontin (OPN)

Osteoprotegerin (OPG)

P-selectin (SELP)

Paraoxonase (PON3)

Peptidoglycan recognition protein 1 (PGLYRP1)

Perlecan (PLC)

Plasminogen activator inhibitor 1 (PAI)

Platelet endothelial cell adhesion molecule (PECAM-1)

Platelet-derived growth factor subunit A (PDGF subunit A)

Proprotein convertase subtilisin/kexin type 9 (PCSK9)

Protein delta homolog 1 (DLK-1)

Pulmonary surfactant-associated protein D (PSP-D)

Resistin (RETN)

Retinoic acid receptor responder protein 2 (RARRES2)

Scavenger receptor cysteine-rich type 1 protein M130 (CD163)

Secretoglobin family 3A member 2 (SCGB3A2)

Spondin-1 (SPON1)

ST2 protein (ST2)

Tartrate-resistant acid phosphatase type 5 (TR-AP)

Tissue factor pathway inhibitor (TFPI)

Tissue-type plasminogen activator (t-PA)

Transferrin receptor protein 1 (TfR)

Trefoil factor 3 (TFF3)

Trem-like transcript 2 protein (TLT-2)

Tumor necrosis factor ligand superfamily member 13B (TNFSF13B)

Tumor necrosis factor receptor 1 (TNF-R1)

Tumor necrosis factor receptor 2 (TNF-R2)

Tumor necrosis factor receptor superfamily member 10C (TNFRSF10C)

Tumor necrosis factor receptor superfamily member 14 (TNFRSF14)

Tumor necrosis factor receptor superfamily member 6 (FAS )

Tyrosine-protein kinase receptor UFO (AXL)

Tyrosine-protein phosphatase non-receptor type substrate 1 (SHPS-1)

Urokinase plasminogen activator surface receptor (U-PAR)

Urokinase-type plasminogen activator (uPA)

von Willebrand factor (vWF)

**Text S3. Standard biomarker measurements**

Plasma NT-proBNP was analyzed using an electrochemiluminescence immunoassay (lower limit of detection, LLD 5 ng/L, Elecsys 2010; Roche Diagnostics, Indianapolis, IN). Cardiac troponin T was also measured using an electrochemiluminescence immunoassay (LLD 3 ng/L, Elecsys 2010 immunoassay analyzer; Roche Diagnostics, Indianapolis, IN). CRP was analyzed using an immunoturbidimetric assay (LLD 0.3 mg/L, Roche Hitachi 912 chemistry analyzer; Roche, Basel, Switzerland). Creatinine was determined by a colorimetric test by the Jaffe’s reaction in undiluted plasma (LLD: 0,14 mg/dl). All coefficients of variation of these four biomarkers were below 5%.

**Text S4. Power calculation and missing data**

The current investigation comprised 250 patients, of whom 66 reached the primary end point. For baseline measurements, these numbers are sufficient to detect odds ratios around 2 for the upper quintile of a biomarker associated with the end point (α error .05, power of 80%) when comparing cases with noncases. For repeated measurements, power is further enhanced. Based on input parameters derived from the benchmark blood biomarker NT-proBNP, and using 500 simulations, we calculated that using 3 measurements per person, a difference in change of NT-proBNP level over time of 10 pmol/L per month can be demonstrated between cases and non-cases (α-error 0.05, power of 80%). This difference is very small in clinical terms, demonstrating that the study has high statistical power.

Data on all variables were complete, except for systolic blood pressure patients and chronic heart failure duration which were missing in <5%. These missing values were imputed using the patients’ clinical and outcome data.

**References**

49. World Health Organization. Classification of Diseases (ICD) [Internet].

50. McMurray JJ, Adamopoulos S, Anker SD, Auricchio A, Bohm M, Dickstein K, Falk V, Filippatos G,Fonseca C, Gomez-Sanchez MA, Jaarsma T, Kober L, Lip GY, Maggioni AP, Parkhomenko A, Pieske BM, Popescu BA, Ronnevik PK, Rutten FH, Schwitter J, Seferovic P, Stepinska J, Trindade PT, Voors AA, Zannad F, Zeiher A, Guidelines ESCCfP. ESC Guidelines for the diagnosis and treatment of acute and chronic heart failure 2012: The Task Force for the Diagnosis and Treatment of Acute and Chronic Heart Failure 2012 of the European Society of Cardiology. Developed in collaboration with the Heart Failure Association (HFA) of the ESC. *Eur Heart J* 2012;**33**:1787-1847.
